# Community-based intervention *via* WeChat official account to improve parental health literacy among primary caregivers of children aged 0 to 3 years: Protocol for a cluster randomized controlled trial

**DOI:** 10.3389/fpubh.2022.1039394

**Published:** 2023-01-06

**Authors:** Yun Li, Qiu-Li Xiao, Mu Li, Yue Zhang, Min Chen, Chun-Hua Jiang, Shu-Rong Kang, Ying Zhang, Jun Huang, Hong Jiang

**Affiliations:** ^1^Department of Child Health Care, Shanghai Minhang Maternal and Child Health Care Hospital, Shanghai, China; ^2^Department of Maternal, Child and Adolescent Health, School of Public Health, Key Lab of Health Technology Assessment, National Health Commission of the People's Republic of China, Fudan University, Shanghai, China; ^3^School of Public Health, The University of Sydney, Sydney, NSW, Australia; ^4^China Studies Centre, The University of Sydney, Sydney, NSW, Australia

**Keywords:** parental health literacy, social media, WeChat account, caregivers, cluster randomized controlled trial

## Abstract

**Background:**

Parental health literacy is an important determinant of children's health, especially during the critical window of early development in the first 3 years. As the information communication technology develops, health education *via* social media is widely used to deliver health information. However, few studies have explored the effect of intervention *via* social media on parental health literacy.

**Objective:**

This study aims to determine whether a WeChat official account-based health intervention can improve parental health literacy of primary caregivers of children aged 0 to 3 years in Minhang District, Shanghai, China.

**Methods:**

The cluster randomized controlled trial includes all 13 community health centers (CHCs) in Minhang District, Shanghai. We take each CHCs as a cluster in the randomization. The CHCs are randomly allocated to the intervention or the control group through random sequence generation. Ninety primary caregivers of children aged 0 to 2 years will be recruited from each CHC, 1170 in total. Caregivers in the intervention group will be provided with a series of video clips and online reading material links on scientific parenting *via* a WeChat account. Caregivers in the control group will receive printed educational materials with similar contents to the intervention group. All the participants will access routine child health care and be followed up for 9 months. Online assessment of health literacy will be conducted for both groups before and after the intervention. The primary outcome is the change in the total scores of parental health literacy using a validated instrument. The data of secondary outcomes, such as exclusive breastfeeding in the first 6 months, anthropometric measurements, and disease conditions, will be extracted from routine health care records. Generalized linear mixed model (GLMM) will be used for data analyses.

**Discussion:**

Compared with traditional health education, health intervention *via* WeChat official account could be a feasible and effective solution to improve parental health literacy.

**Trial registration:**

This trial is registered with the Chinese Clinical Trial Registry (ChiCTR): (#ChiCTR2000031711) on April 07, 2020.

## 1. Introduction

The “First Thousand Days” refers to the significant period from conception to the child's second birthday (1). During the first 3 years after birth, the quality of nurturing and care by caregivers plays an essential role in promoting children's immediate health and laying the foundation of their lifelong health (2). A growing body of evidence shows that parents' health literacy was closely associated with parenting practices. Children whose parents had low parental health literacy were more likely to experience poorer health outcomes, such as poor glycemic control (3), poor asthma control (4), and high emergency care utilization (5), especially for younger children. Low parental health literacy was also related to adverse health care behaviors, such as not practicing breastfeeding (6), incorrectly administering prescribed medicine (7), and allowing children exposed to more than 2 h television (8). However, limited interventions have been implemented to improve parental health literacy worldwide.

Health literacy, defined by the World Health Organization European Region (EU WHO) in 2013, is a multi-dimensional concept, defined as competencies of accessing, understanding, appraising, and applying health-related information within each of three health domains including health care, disease prevention, and health promotion (9). This conceptual model identifying twelve subdimensions can serve as a framework for developing measures and intervention strategies for enhancing health literacy. Based on this model, the Chinese Parental Health Literacy Questionnaire of Children 0 to 3 Years old (CPHLQ) was developed to assess parental health literacy (10, 11). By now, most studies just focused on single parenting knowledge or behavior and very few intervention studies have been carried out to improve overall parental health literacy among caregivers based on the integrated theoretical model.

With the rapid development of information communication technology, social media, such as Facebook, Twitter, and Instagram, has become significant channels and tools for information acquisition and exchange (12–16). WeChat, a free Chinese multi-purpose messaging and social media application, is the most widely and frequently used social media platform in China. It has monthly attracted more than 1.2 billion active users worldwide by the end of 2020 (17). WeChat official accounts (WOAs), a new function module of WeChat, can be used freely by governments, enterprises, and organizations to disseminate information and interact with their subscribers *via* text, image, audio, and video (18). In 2020, there were more than 360 million people acquiring information daily from WOAs (17).

WeChat has been increasingly used for disseminating health information. A recent national survey from 32 provinces and cities across China (19) found that one-third of participants regularly obtained health information through WeChat, and nearly two-thirds of participants reportedly expected to access health education from WOAs. The survey results also revealed that health education *via* WeChat and WOAs was convenient, timely, cost-effective, and protective of privacy. Substantial intervention programs *via* WeChat have proven to be effective and feasible in enhancing health literacy. For instance, a WOA-based intervention proved an effective and well-accepted strategy for improving malaria health literacy among Chinese expatriates in Niger (20). The results of a large-sample intervention in Sichuan, province of China, showed that WeChat platform helped to improve residents' health literacy and promote the development of healthy lifestyle (21). Health interventions *via* WeChat also have been shown with the potential to promote the satisfaction, accessibility, and convenience of child health care services (22), improve health management, and reduce medical cost (23).

Given the wide application of WOAs (24), we propose to conduct a cluster randomized controlled trial to determine the effectiveness of a WOA-based health intervention for improving the health literacy of primary caregivers with children aged 0 to 3 years in communities of Minhang District, Shanghai, China.

## 2. Objective

This study aims to develop an intervention program to improve parental health literacy among primary caregivers of children aged 0 to 3 years *via* WOA with clustered randomized controlled trial design in all 13 communities of Minhang District, Shanghai, China. The specific objectives of this cluster randomized controlled trial are:

(1) To develop a community-based health intervention program based on the integrated theoretical model of health literacy defined by EU WHO.
(2) To implement the intervention among primary caregivers of children aged 0 to 3 years in the intervention group in communities of Minhang District, Shanghai, China.
(3) To determine whether the WOA-based intervention is more effective in improving caregivers' health literacy, health-related behaviors, and health outcomes of the children compared with traditional health education.

## 3. Materials and methods

### 3.1. Overall study design

**Figure 1:**
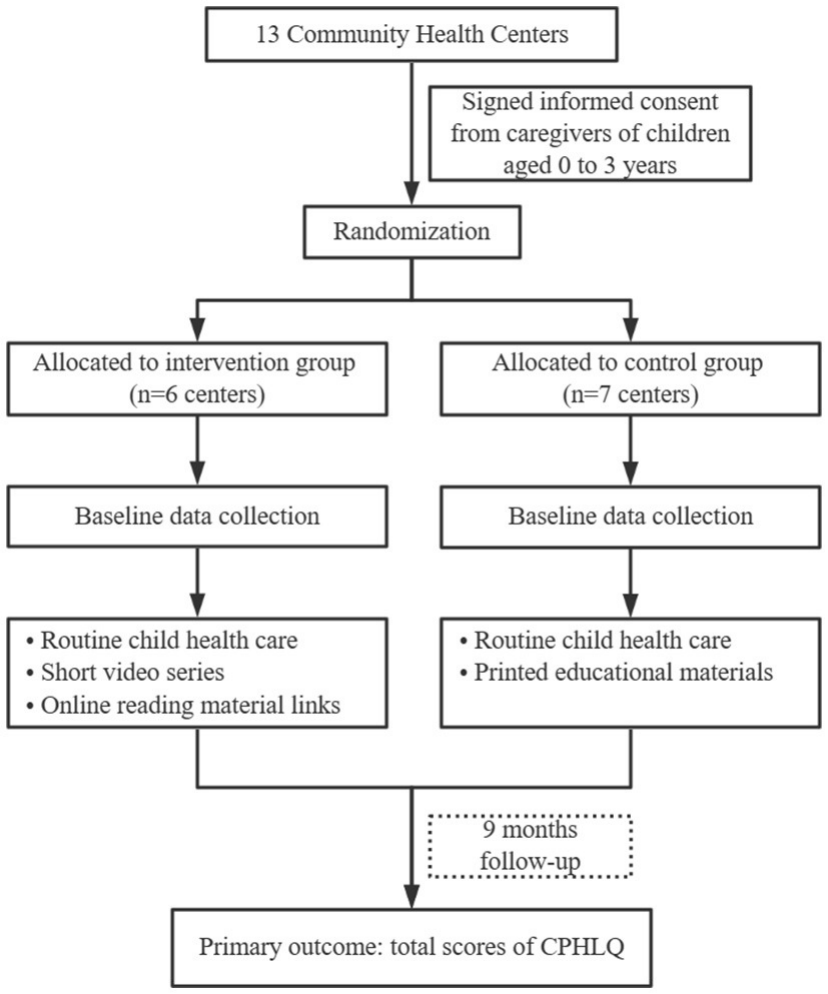
Research flow chart.

This cluster randomized controlled trial (cRCT) will be conducted in all 13 community health centers (CHC) of Minhang District in Shanghai. Each CHC will be designated as a cluster in the randomization. Prior to the participant recruitment, 13 CHCs will be randomized into the intervention group or the control group through random sequence generation. Eligible caregivers will be recruited after informed consent is obtained. Interventions will be implemented at the community level rather than the individual level to minimize contamination among participants within the same community. All participants will be followed up for 9 months (see Figure 1). We will compare the changes in scores of parental health literacy between the two groups before and after the intervention.

Ethical approval to conduct this trial has been granted by the Ethics Committee of Shanghai Minhang District Maternal and Child Health Hospital (#[2020]KS-01). The research is registered with the Chinese Clinical Trial Registry (#ChiCTR2000031711).

### 3.2. Study setting

The study will be conducted in Minhang district, Shanghai, China. Minhang district is located in the southwest of Shanghai, with 7,000 newborns annually and 12,760 children aged 0–3 years in 2020. A previous survey showed that the health professionals in Minhang district were facing huge gaps between limited health workforce and high demand for child care services (25). All 13 CHCs of Minhang district will be included in the study. Each CHC, where routine child health care services are provided for all children in the catchment area, will be a cluster.

### 3.3. Blinding

Due to the nature of the intervention, service providers and participants will not be blinded to the group allocation. Immediately after the baseline data collection, group allocation is revealed to participants. The statistician will be masked to group allocation during data analyses.

### 3.4. Participants and recruitment

Since the intervention will last 9 months and parental health literacy questionnaire applies to children only under 3 years, we will recruit primary caregivers of children aged 0 to 2 years at baseline. In this study, primary caregiver is defined as the person providing the major care for a child, including parents, grandparents, and other caregivers like nanny.

Recruitment will be conducted among caregiver-child dyads attending child health care in the child health clinics of CHCs. All study participants will complete a written informed consent before the randomization allocation is revealed. Once eligibility is established and consent obtained, caregivers will be invited to subscribe to the WeChat account for data collection or/and intervention delivery.

#### 3.4.1. Inclusion criteria

(1) Primary caregivers of children aged 0 to 2 years old.
(2) Those who are above the third grade of primary school and able to communicate normally in oral and written form.
(3) Voluntary participation.
(4) Plan to live in the recruitment areas for at least a year.

#### 3.4.2. Exclusion criteria

(1) Not primary caregivers of children aged 0 to 2 years old.
(2) Illiterate and unable to communicate in the form of speaking and writing.

### 3.5. Randomization and allocation concealment

All 13 CHCs in Minhang district will be randomly allocated to either the intervention or control groups through random sequence generation. In addition, group allocation will not be revealed to participants until they provide their written informed consent.

### 3.6. The WOA-based intervention program

#### 3.6.1. Intervention group

The WeChat account called Scientific Parenting is developed to conduct twice online evaluation and the 9-month intervention. Caregivers in both intervention and control groups will be asked to subscribe to and register with the account by entering information on their relationships with the child, the CHCs they belong to, and their children's birthday and unique health record number. Caregivers in the control CHCs cannot access the online intervention, which minimizes potential contamination caused by direct information sharing within the same CHC.

**Figure 2:**
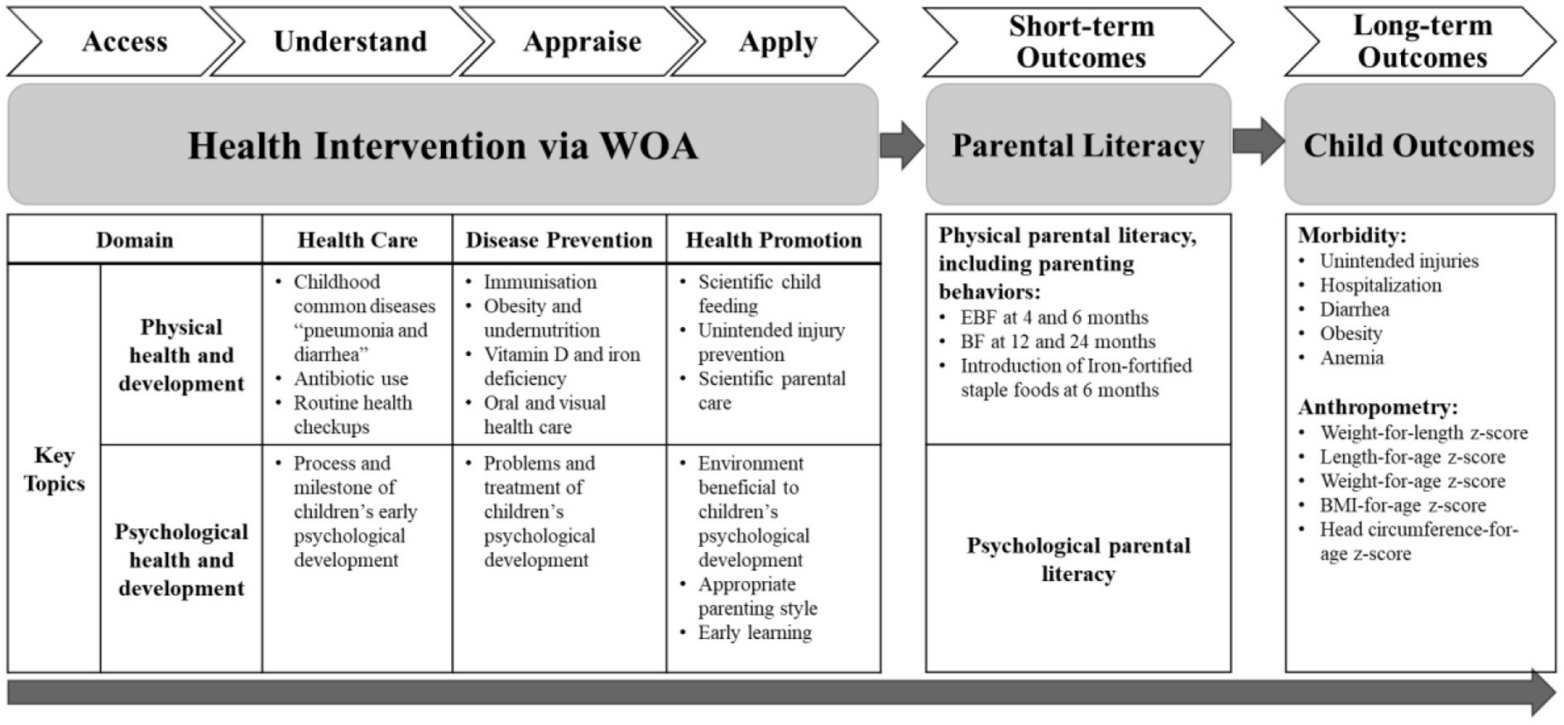
A logic model for the WOA-based intervention.

The intervention content was designed according to 12 sub-dimensions of health literacy defined by EU WHO (9). Based on the framework of health literacy, 15 key topics about children's health and development were generated and allocated to three health domains “health care, disease prevention, health promotion,” 10 in physical and 5 in psychological part through literature review and expert consultation. For example, there are physical and psychological health and development parts in health care domain. The physical health and development part includes three key topics of childhood common diseases “pneumonia and diarrhea,” antibiotic use, and routine health checkups (see Figure 2). The intervention contents of each key topic were developed to improve caregivers' capacity of accessing, understanding, appraising, and applying child health care information. Based on the key topics, the intervention materials include the following modules: essential information in child health care, neonatal nursing skills, neonatal health problems and management, scientific feeding guidance, childhood obesity prevention, child pneumonia identification, unintended injury prevention, hand-foot-mouth disease prevention, appropriate handwashing method, psychological problems and warning signs, early symptoms of autism, children physical activity guidance, children physical development and scientific feeding, guidance for parenting, and parent-child interaction games (see Supplementary Table S1).

Once subscribed to the account, caregivers in the intervention group will receive all the online health materials at once. They can self-navigate, select topics they are interested in, decide the order of material watching, and can access the intervention materials at their own pace. The online materials consist of two types of materials: video materials and online reading material links. The video materials include 4 video clips of experts' lecture each lasting 13 to 33 min and 6 animated video clips each lasting 1 to 5 min, with a total duration of 2 h. They could be watched repeatedly and the number of times of watching will be counted and recorded by the platform in each user's account. The caregivers in the intervention group can also access online reading materials related to scientific parenting through the provided links, such as e-books, websites, and other WeChat public accounts. Through this WOA-based intervention, participants in the intervention clusters can access reliable parental health information from a trusted official source. Supplementary Table S1 details the WOA-based health intervention program.

#### 3.6.2. Control group

Caregivers in the control group will be provided with printed educational materials with similar contents as the video materials delivered to the intervention group, after recruitment. Caregivers of the control group can decide the orders of material reading according to their priority needs and interests. Supplementary Table S2 details the printed materials the control group will receive.

In addition, the same routine child health care services, including anthropometric measurements of weight and length, inquiry about feeding practice and dietary intake, and assessment of disease conditions will be provided to the intervention group and the control group (22).

### 3.7. Sample size and power calculation

The sample size calculation was based on detecting the changes in primary outcome, the total scores of parental health literacy after 9-month intervention between the intervention and the control group. We assume that the intra-cluster correlation coefficient is 0.05 and that the coefficient of variation is 0.2 according to a previous survey (10). Based on this assumption, 949 participants (73 in each CHC) are needed to detect a difference of 15 in total score between the intervention and the control groups at 0.05 significance level, 90% power. Given an estimated lost follow-up rate of 20%, a total of 1,183 (91 in each CHC) primary caregivers with children aged 0 to 2 years at the recruitment will be needed. All sample size and power calculations were performed using the PASS software (Power Analysis and Sample Size, Version 15, NCSS, Georgia).

### 3.8. Data collection

The primary outcome of the program is the change in total scores of parental health literacy, which will be assessed by a validated questionnaire, the Chinese Parental Health Literacy Questionnaire of Children 0 to 3 years old (CPHLQ) (10, 11). The CPHLQ was established within the framework of integrated theoretical model of health literacy developed by EU WHO (9) and has been shown with good reliability and validity among primary caregivers of children under 3 years across different regions in China (10, 11, 26). This questionnaire consists of a 39-item subscale for physical health literacy and a 35-item subscale for psychological health literacy, the Cronbach's alpha of which is 0.915 and 0.918, respectively (26). The physical subscale focuses on health literacy toward scientific child feeding, unintended injury prevention, scientific parental care, obesity and undernutrition, Vitamin D and iron deficiency, oral and visual health care, immunization, routine health checkups, antibiotic use, and common childhood diseases “pneumonia and diarrhea.” The psychological subscale focuses on process and milestone of children's early psychological development, problems and treatment of children's psychological development, environment beneficial to children's psychological development, appropriate parenting style, and early learning. Both the physical and psychological part use a percentage grading system, with full score of 100 for each part and a total score of 200.

Secondary outcomes will include: (1) the awareness rate of vitamin D (VD) supplementation after birth; (2) parenting behaviors such as the rate of exclusive breastfeeding (EBF) in the first 4 and 6 months, the rate of breastfeeding in the first 12 and 24 months, and introduction of Iron-fortified staple foods at 6 months; (3) anthropometric measurements of children such as weight-for-length z-score (WHZ), length-for-age z-score (HAZ), weight-for-age z-score (WAZ), BMI-for-age z-score (BAZ), and head circumference-for-age z-scores(HCZ); (4) health outcomes of children such as the incidence of unintended injuries, hospitalization, diarrhea, obesity, and anemia.

Both baseline and final evaluation at the end of nine months survey will be conducted online *via* the aforementioned WOA. Besides collecting data for the outcome measures, we will collect demographic information of the caregiver-child dyads. We will also evaluate participants' engagement of the intervention through their completion rate of video clips and number of times of video watching, which both can be automatically tracked by a functional module designed by the research team on WOA platform.

#### 3.8.1. At baseline

In the baseline survey, demographic information will be collected *via* online questionnaire survey, including caregiver's relationship with the child, education level, source of parental information access, family income, number of children, child's age, gender, and Hukou [the Chinese official residency registration by location, which is directly related to social welfare and administration (27, 28)]. Then an assessment on parental health literacy will be conducted using the CPHLQ. In addition, we will collect parents' self-reported priority needs on parental health literacy, through a multiple-choice question with options involving the topics included in CPHLQ.

#### 3.8.2. At 9 months

After nine-month intervention, online assessment will be conducted again in both intervention and control groups. We will compare the changes of scores on parental health literacy of the two groups to evaluate the efficacy of the WOA-based intervention. We will also collect information on participants' awareness of VD supplementation, parenting behaviors including routine checkup of child's vision and oral health, and children's health outcomes such as occurrence of unintended injury, hospitalization, and diarrhea.

Data on exclusive breastfeeding, breastfeeding, timing of introducing solid foods, anthropometry, and disease conditions such as obesity and anemia, will be extracted from health records from CHCs, which will be collected and recorded by doctors as part of a routine child health checkups (29). Anthropometric measurement will be conducted using established methods and a standard protocol (30).

### 3.9. Data management

Data will be kept anonymous in a server that supports the instant update and confidentiality, and will be used only for this study. Data will be password protected and only authorized research team members will have the access to the links of the database.

### 3.10. Data analysis plan

Descriptive statistic analysis will be performed for all outcomes. For continuous variables, means will be compared using *t*-tests, or non-parametric equivalents for non-normally distributed variables. For categorical variable, chi-squared tests will be used. WHZ, HAZ, WAZ, BAZ, and HCZ will be calculated using WHO Anthro v3.2.2, as the World Health Organization suggested (31).

Generalized linear mixed model (GLMM) will be used for assessing intervention effects on parental health literacy. Covariates will include demographic characteristics and baseline scores. Each CHC will work as a single random effect. This model controlling for the correlation between baseline and follow-up values will not require a random effect for time or a Time × Group interaction term to interpret intervention effects (32). Additionally, given the relatively small number of clusters, the Satterthwaite correction will be used in GLMM to maintain an appropriate Type I error (33). The Satterthwaite correction could achieve an approximation of the degree of freedom according to the residual variances at different levels of each variable (34). The same method will be used for the analyses of secondary outcomes such as parenting behaviors, children's growth, and health outcomes.

All analyses will be conducted with “intention to treat” analysis and multiple imputation will be used to impute the missing entries based on participants' demographics. Results with a Type I error rate of *p* < 0.05 in two-sided tests will be considered statistically significant. All analyses will be performed using SPSS software for Windows (version 22.0; SPSS Inc.) and R Statistical Software (version 4.1.2).

### 3.11. Data monitoring plan

To ensure the reliability of data collection, all staffs from 13 CHCs will receive unified training with the same protocol and quality controllers in the district maternal and child health institute will check the data everyday.

## 4. Discussion

Parental health literacy is an important determinant of children's health. In previous study, intervention strategies aiming to improve parental knowledge have been explored. A perioperative health education *via* WeChat platform effectively enhanced parents' knowledge of care for children with heart diseases (22). A WeChat-based parenting training proved a promising training method for improving parenting attitude among mothers of children with autism during the COVID-19 pandemic (35). A WeChat health education in Qinghai, China, significantly increased exclusive breastfeeding rate in early life (36). However, none of them developed interventions based on the integrated conceptual model of health literacy by EU WHO which includes three health domains (health care, disease prevention, and health promotion) and four competences of information processing (accessing, understanding, appraising, and applying) for each domain (9).

To our knowledge, this will be the first cRCT of using WeChat to comprehensively improve parental health literacy among primary caregivers with children under 3 years. Since across the world, many primary health facilities are suffering from limited medical human resources and competencies to provide quality health education, there is an urgent need to explore feasible and effective approaches to deliver health information and supports to caregivers of young children (25). Compared with traditional medium, social media has been applied widely for its valuable features such as low cost, large user base, various formats of information, and rapid and convenient information delivery and communication (37).

There are several limitations in this study. First, our intervention does not involve environment or policy change to improve the parental health literacy. In the future, expanded intervention dimension is needed to further enhance the intervention effect. Second, all the online materials will be delivered to the participants at once after the recruitment. This one-intervention-for-all strategy may not be the best to meet individualized needs. Third, the outcome measures do not include psychological development due to the increased burden to caregivers. In addition, psychological assessment is not included in the routine child health checkup and needs specialized training on health care staffs. Fourth, since this study will be carried out in all communities in one district of Shanghai, generalizability to non-urban locations or populations with diverse demographics might be limited.

This intervention study can provide primary caregivers of young children with reliable parental health information from a trusted official source. If the intervention proves effective, it will have great potential for the application in other areas with limited health resources to improve health literacy and health outcomes. This type of intervention may also alleviate the imbalance in child health care services between different regions and socio-economic groups, thus greatly impacting on health inequities among children.

## Ethics statement

The studies involving human participants were reviewed and approved by the Ethics Committee of Shanghai Minhang District Maternal and Child Health Hospital (approval number #[2020]KS-01). Written informed consent to participate in this study was provided by the participants' legal guardian/next of kin.

## Author contributions

YL, JH, and HJ conceptualized and designed the study, contributed to the development of the trial, and obtaining the funding. Q-LX, YL, and HJ drafted the manuscript. ML provided critical comments and revisions. All authors read and approved the final manuscript.
