# Supplementary material for: Community-based intervention via WeChat official account to improve parental health literacy among primary caregivers of children aged 0 to 3 years: Protocol for a cluster randomized controlled trial
Source: Front Public Health. 2023 Jan 6;10:1039394. doi: 10.3389/fpubh.2022.1039394 (PMC9853903; doi:10.3389/fpubh.2022.1039394)
Supplement: Supplementary file 1 [file Table_1.docx]

**Module summaries for the WeChat official account-based intervention program**

Table 1 Appendix for the WeChat official account-based intervention

| Module title | Key topics | Main contents | Type and duration |
| --- | --- | --- | --- |
| Essential information of health care for children aged 0 to 3 years | - Scientific child feeding - Immunisation - Routine health checkups - Obesity and undernutrition - Vitamin D and iron deficiency - Common childhood diseases - Unintended injury prevention | - Scientific child feeding guidance for different age - Encouragement for responsive child feeding - Cautions and common adverse reactions of immunisation - Characteristics of children physical and psychological development - Encouragement for routine health checkup and disease screening - Prevention of common health problems including obesity, undernutrition, and anemia - Recommendation for supplementation of vitamin A and D as needed - Prevention and early identification of common diseases such as pneumonia and diarrhea - Prevention of unintended injuries | 33-minute video clip of experts’ lecture |
| Neonatal nursing skills | - Immunisation - Scientific parental care | - Cautions for immunisation - Tips for neonatal care such as thermal care, and cord hygiene - Identification of common problems such as pseudo menstruation, hydrocele, and common skin problems - Characteristics of children growth and development - Recognition of neonatal health problems from feces | 22-minute video clip of experts’ lecture |
| Neonatal health problems and management | - Routine health checkups - Scientific parental care | - Recommendation for neonatal hearing screening - Identification of common physiological signs such as mammary glands, pseudo menstruation, hydrocele, horse tooth, and common skin problems - Recognition of warning signs of child illness such as breast milk jaundice, hypoglycemia, fever, infection, and vomiting | 31-minute video clip of experts’ lecture |
| Scientific feeding guidance | - Scientific child feeding - Vitamin D and iron deficiency - Scientific parental care | - Exclusive breastfeeding guidance for infants aged 0 to 6 months - Feeding guidance for infants aged 7 to 12 months - Encouragement for responsive child feeding and growth monitoring - Encouragement for developing healthy eating habits - Recommendation for appropriate dose for vitamin D supplementation and sunlight exposure | 2-minute animated video clip |
| Childhood obesity prevention | - Obesity and undernutrition | - Contributing factors to childhood obesity - Prevention and control of childhood obesity - Encouragement for growth monitoring and physical activity | 2-minute animated video clip |
| Child pneumonia identification | - Childhood common diseases “pneumonia and diarrhea” | - Early identification of Child pneumonia | 2-minute animated video clip |
| Unintended injury prevention | - Unintended injury prevention | - Definition and awareness of risky circumstances of unintended injuries - Prevention of unintended injuries | 2-minute animated video clip |
| Hand-foot-mouth disease (HFMD) prevention | - Childhood common diseases | - Causes, transmission route, and prevention of HFMD | 2-minute animated video clip |
| Appropriate handwashing method | - Scientific parental care | - Benefits, critical times, and appropriate steps of handwashing | 2-minute animated video clip |
| Psychological problems and warning signs | - Problems and treatment of children’s psychological development - Process and milestone of children’s early psychological development | - Characteristics of language, motor, and social interaction development at different age - Recognition of warning signs of abnormal psychological at different age | 13-minute video clip of experts’ lecture |
| Early symptoms of autism-1 | - Problems and treatment of children’s psychological development | - Definition and symptoms of childhood autism | 4-minute animated video clip |
| Early symptoms of autism-2 | - Problems and treatment of children’s psychological development | - Development, significance, and entries of the CHAT-23 scale | 5-minute animated video clip |
| Children physical activity guidance | - Scientific parental care | - Encouragement for physical activity - Physical activity guidance for children aged 0 to 5 years | Links to book |
| Children physical development and scientific feeding | - Scientific child feeding | - Dietary Guidelines for Chinese women and children (2016 version) - Feeding Guidelines for Preterm Infants | Links to book |
| Guidance for parenting | - Scientific parental care - Appropriate parenting style - Early learning - Environment beneficial to children’s psychological development | - Guidelines for Preterm Infants Parenting - Parenting Tips by Shrimp Mommy | Links to book |
| Parent-child interaction games | - Appropriate parenting style - Early learning | - Positive Discipline - Joyful Parent-child Interaction Games - Game Guidance for Children under 6 Years | Links to book |
| Relevant public accounts | - Common childhood diseases - Routine health checkups - Scientific child feeding - Unintended injury prevention - Scientific parental care - Early learning - Process and milestone of children’s early psychological development - Problems of children’s Psychological development | - Dr. Duan (providing guidance for complementary feeding, motor development, growth monitoring, and oral health care) - Dr. Pei (providing searching, livestreaming, and counselling parental service) - Dr. Yu (providing tips for parenting at different age phases and common pediatric diseases) - Dr. Bao (providing tips for language and motor development, growth monitoring, and psychological assessment) - Pediatrician (providing online support group service and guidance for physical and psychological development and parent-child reading) - Dr. Huang (providing guidance for common digestive diseases, medication, and unintended injuries) - Dingxiang Mommy (providing parenting information searching and language development assessment service) | Links to WOA |
| Parenting app by Shanghai Hyd Light Education Technology Company | - Scientific child feeding - Routine health checkups - Immunisation - Early learning - Problems of children’s psychological development | - Guidance for breastfeeding and home nurturing - Encouragement for routine health checkup and immunisation - Encouragement and guidance for parent-child games - Recognition of warning signs of children psychological problems | Link to app |
| The Scientific Parenting site | - Scientific child feeding - Early learning | - Encouragement and guidance for breastfeeding, parent-child interaction game, early childhood learning, and gender education | URL: http://www.kna-tech.com/ |
