# Supplementary material for: Community-based intervention via WeChat official account to improve parental health literacy among primary caregivers of children aged 0 to 3 years: Protocol for a cluster randomized controlled trial
Source: Front Public Health. 2023 Jan 6;10:1039394. doi: 10.3389/fpubh.2022.1039394 (PMC9853903; doi:10.3389/fpubh.2022.1039394)
Supplement: Supplementary file 2 [file Table_2.docx]

**Summaries for printed educational materials disseminated to the control group**

Table 2 Appendix for printed educational materials disseminated to the control group

| Topics | Main contents |
| --- | --- |
| Common childhood diseases | - Early identification of child pneumonia - Transmission and prevention of hand-foot-mouth disease - Appropriate handwashing methods - Benefits, critical times, and appropriate steps of handwashing |
| Routine health checkups | - Recommendation for routine child’s oral check |
| Immunisation | - Cautions and common adverse reactions of immunisation |
| Obesity and undernutrition | - Contributing factors to obesity and undernutrition - Prevention of childhood obesity and undernutrition - Short-term and long-term impacts of childhood obesity - Encouragement for growth monitoring and physical activity - Encouragement for responsive feeding and balanced diet - Encouragement for developing healthy eating habits - Tips for picky eating |
| Vitamin D and iron deficiency | - Recommendation for supplementation of vitamin A and D as needed - Recognition of warning signs of iron-deficiency anemia - Recommendation of dietary intaking of Iron-rich food - Tips of supplementation and storage of Iron-fortified food |
| Scientific child feeding | - Exclusive breastfeeding guidance for infants aged 0 to 6 months - Feeding guidance for infants aged 7 to 12 months - Encouragement for responsive child feeding and balanced diet |
| Unintended injury prevention | - Definition and prevention of children unintended injuries |
| Physical activity | - Recommendation for childhood physical activity - Guidance for physical activity |
| Environment beneficial to children's psychological development | - Necessity and encouragement of healthy home environment - Encouragement for child involvement |
| Appropriate parenting style | - Rules and limits of children behaviors - Guidance for reinforcements and groundings, such as praise, rewards, and criticism |
| Early learning | - Encouragement for parent-child interaction games - Encouragement for establishment of reading habits |
| Process and milestone of children’s early psychological development | - Characteristics of language, motor, and social interaction development at different age - Recognition of warning signs of language and motor problems - Prevention of children anxiety and fear - Tips for psychological development promotion |
| Problems of children's psychological development | - Recognition of warning signs of psychological and behavioral abnormalities |
